# Construction and Characterization of Synthetic Bacterial Community for Experimental Ecology and Evolution

**DOI:** 10.3389/fgene.2018.00312

**Published:** 2018-08-14

**Authors:** Johannes Cairns, Roosa Jokela, Jenni Hultman, Manu Tamminen, Marko Virta, Teppo Hiltunen

**Affiliations:** ^1^Department of Microbiology, University of Helsinki, Helsinki, Finland; ^2^Department of Biology, University of Turku, Turku, Finland

**Keywords:** microbial community, model system, synthetic ecology, experimental evolution, whole-genome sequencing

## Abstract

Experimental microbial ecology and evolution have yielded foundational insights into ecological and evolutionary processes using simple microcosm setups and phenotypic assays with one- or two-species model systems. The fields are now increasingly incorporating more complex systems and exploration of the molecular basis of observations. For this purpose, simplified, manageable and well-defined multispecies model systems are required that can be easily investigated using culturing and high-throughput sequencing approaches, bridging the gap between simpler and more complex synthetic or natural systems. Here we address this need by constructing a completely synthetic 33 bacterial strain community that can be cultured in simple laboratory conditions. We provide whole-genome data for all the strains as well as metadata about genomic features and phenotypic traits that allow resolving individual strains by amplicon sequencing and facilitate a variety of envisioned mechanistic studies. We further show that a large proportion of the strains exhibit coexistence in co-culture over serial transfer for 48 days in the absence of any experimental manipulation to maintain diversity. The constructed bacterial community can be a valuable resource in future experimental work.

## Introduction

Testing ecological and evolutionary theory in a highly controlled manner using simple laboratory setups with one or two microbial species (Fraser and Keddy, 1997; Buckling et al., 2009) has produced important insights into ecological interactions–e.g., competition, cooperation, and cross-feeding interactions (Helling et al., 1987; Treves et al., 1998; Rozen and Lenski, 2000; Shou et al., 2007; Harcombe, 2010); the role of cheaters (MacLean and Gudelj, 2006); predator–prey interactions (Shertzer et al., 2002); and host–parasite interactions (Morgan et al., 2005)–and evolutionary processes–e.g., the evolution of coexistence (Good et al., 2017), coevolution between species (Hall et al., 2011; Brockhurst and Koskella, 2013), and eco-evolutionary feedback dynamics (Yoshida et al., 2003; Hiltunen and Becks, 2014). However, there is an increasing awareness that ecological and evolutionary processes can be fundamentally altered in more complex multispecies communities owing to several features such as altered competitive interactions and multiple selection pressures (Dunham, 2007). Recent empirical findings show, for example, that pairwise interactions can be strongly altered in the presence of other species (Kastman et al., 2016) and the rate of adaptation of species can differ between monocultures and communities (Lawrence et al., 2012). Even a basic understanding of certain characteristics of microbial life such as horizontal gene transfer (Smillie et al., 2011), metabolic interactions and spatial heterogeneity (Elias and Banin, 2012; van Gestel et al., 2014) requires investigation of multispecies settings integral to them. Furthermore, several key ecological features are specific to multispecies communities, such as diversity, stability, succession and high-order (e.g., four-way) species interactions (Bairey et al., 2016). There is therefore a profound need to expand the biotic complexity of study systems used in the fields of experimental microbial ecology and evolution.

The design of multispecies model communities in experimental ecology and evolution is part of the emerging field of synthetic ecology where synthetic communities are used for a plethora of basic and applied purposes. Several research attempts have sought mechanistic understanding of specific natural systems, such as methane consuming communities (Yu et al., 2017), plant root colonizing bacteria (Lebeis et al., 2015), the human gut microbiota (Goodman et al., 2011), and cheese rind communities (Wolfe et al., 2014), by complementing observational findings with findings from controlled *in vitro* or *in vivo* studies using synthetic communities. These studies focus on designing synthetic communities that capture the essential features of the natural system being investigated. A typical approach is to determine the prevalent taxa in the natural system or in the core microbiome (Shade and Handelsman, 2012) common to similar systems, to construct a synthetic community of taxonomically representative strains isolated from the natural system, and to culture the community in conditions mimicking the natural system. The limitations of this approach include the potentially important role in ecological functions or evolutionary processes of low-abundance taxa (Liu et al., 2017), microdiversity (Chase et al., 2017), or interactions between bacteria and members of other taxonomic groups such as viruses, unicellular eukaryotic predators or fungi, as well as technical difficulties in mimicking natural conditions in the laboratory (Wolfe, 2018). Likely owing to such limitations, among these studies, cases have been observed both where simple synthetic communities representing predominant taxa in natural systems recapitulate the dynamics in natural systems (Goodman et al., 2011; Wolfe et al., 2014; Lebeis et al., 2015) and where major differences are observed between synthetic and natural systems (Yu et al., 2016).

Compared with studies employing synthetic communities to understand specific natural systems, more applied studies focusing, among others, on medical therapeutics (Petrof et al., 2013; Sheth et al., 2016), bioremediation (Dejonghe et al., 2003; Zomorrodi and Segre, 2016), or biofuel production (Wang et al., 2015), rely even more heavily on a detailed understanding of the characteristics and functions of specific bacterial taxa in natural systems to engineer communities that can successfully perform desired functions. In contrast, studies attempting to investigate highly general ecological or evolutionary processes, similar to traditional experimental microbial ecology and evolution using one- or two-species model systems, do not necessarily seek to, or prefer simple culture conditions over the ability to, accurately represent a particular natural community. For instance, a synthetic community of 72 bacterial strains isolated from tree-hole bacterial communities–but limited to aerobic heterotrophs cultivable in simple laboratory conditions–has been used to investigate several key ecological questions, including the relationship between diversity and ecosystem productivity (Bell et al., 2005) and the success of multispecies invasions during different stages of ecological succession (Rivett et al., 2018). Celiker and Gore (2014), in turn, used a completely synthetic model community comprising six apparently random soil bacterial strains from culture collections to examine the repeatability of change in community composition over time.

In simplified synthetic communities, verisimilitude is sacrificed to obtain relative ease of analysis and modeling, and control of species interactions, non-linear effects from added traits and strains, and evolutionary change (Widder et al., 2016). There is ongoing debate about the utility of simple microcosm setups to understand ecological and evolutionary phenomena (Carpenter, 1996, 1999; Fraser and Keddy, 1997; Drenner and Mazumder, 1999; Benton et al., 2007; Buckling et al., 2009), yet the approach continues to produce major scientific discoveries (van Houte et al., 2016; Good et al., 2017; Betts et al., 2018; Frickel et al., 2018). Similarly, the definition of, need for, and necessary level of representativeness of synthetic communities remain matters of debate (Dolinsek et al., 2016; Widder et al., 2016; Zomorrodi and Segre, 2016; Wolfe, 2018), and are likely strongly dependent on the research questions. Although debated, completely synthetic communities composed of strains isolated from different habitats can also be used to study general questions as well as having special use in studying questions such as community assembly and the evolution of coexistence in newly formed communities.

In this context, the detailed, mechanistic understanding of simpler, less representative synthetic communities can be thought to inform, or even be a prerequisite to, understanding more complex synthetic systems, and ultimately, natural systems. It has been argued that such efforts should focus on understanding a limited set of well-defined model synthetic communities, which would make results comparable between studies and allow collaborative efforts toward mechanistic understanding (Widder et al., 2016). However, not many such systems exist to our knowledge despite the general boom in synthetic ecology. Furthermore, the highly general level studies that exist primarily focus on simple phenotypic analyses (Bell et al., 2005; Foster and Bell, 2012; Celiker and Gore, 2014; Rivett et al., 2016, 2018; Rivett and Bell, 2018), although high-throughput molecular methods, such as amplicon sequencing, (meta)genomics, (meta) transcriptomics, proteomics and metabolomics, which have been promisingly utilized in studies focusing on synthetic systems mimicking natural systems (Goodman et al., 2011; Wolfe et al., 2014; Lebeis et al., 2015; Stopnisek et al., 2016; Yu et al., 2017), could provide valuable insights into the mechanisms behind observed phenotypic and community level features.

To address these needs, we here constructed a simplified experimental system comprising a completely synthetic community of 33 bacterial strains representing two phyla and six classes that can be cultured individually and in co-culture in highly simple laboratory conditions, and that can be individually resolved based on amplicon sequencing. Furthermore, we present draft-level whole genome sequence data as well as information regarding genomic features and phenotypic traits for all strains in the community, facilitating further mechanistic studies. We also present proof of concept for coexistence of a large proportion (14/33) of the strains in co-culture after serial passage of cultures for 48 days. Such a community can be a highly useful resource for experimental microbial ecology and evolution. For instance, we recently used a closely related model system to track the mobility of antibiotic resistance genes in a complex bacterial community (Cairns et al., 2018). For future studies, we envision, for example, exploration of the trajectories of ecosystem composition and genetic structure in response to environmental perturbations or variability in functional trait space. We are also considering using the community as an internal control for improving high-throughput microbial single cell genome sequencing techniques such as epicPCR (Spencer et al., 2016) or metagenome assemblies.

## Materials and Methods

### Constructing Model Community

Strains from the University of Helsinki culture collection (HAMBI) representing diverse taxa were initially screened for the ability to grow individually at 28°C in two complex liquid media: the nutrient-rich proteose peptone yeast extract (PPY: 20 g proteose peptone and 2.5 g yeast extract in 1 l deionized H_2_O) medium or a custom lower-nutrient-level medium containing M9 salt solution and King’s B (KB) nutrients at a 1% concentration compared with full-strength medium (concentrations used: 0.2 g peptone number 3 and 0.1 ml of 85% glycerol in 1 l of dH_2_O), and 0.2 g l^-1^ protozoan pellets (Carolina Biological Supply Co., Burlington, United States). Protozoan pellets were prepared by dissolving in dH_2_O, bringing to boil and filtering through 40 μm to remove particulate matter. Notably, the strains were not selected based on being representative of any particular natural system but rather as a collection of strains, each representing a different taxonomic group, that can grow in simple, uniform laboratory conditions and therefore be easily used to test general ecological and evolutionary theory or techniques (Wolfe, 2018).

**FIGURE 1:**
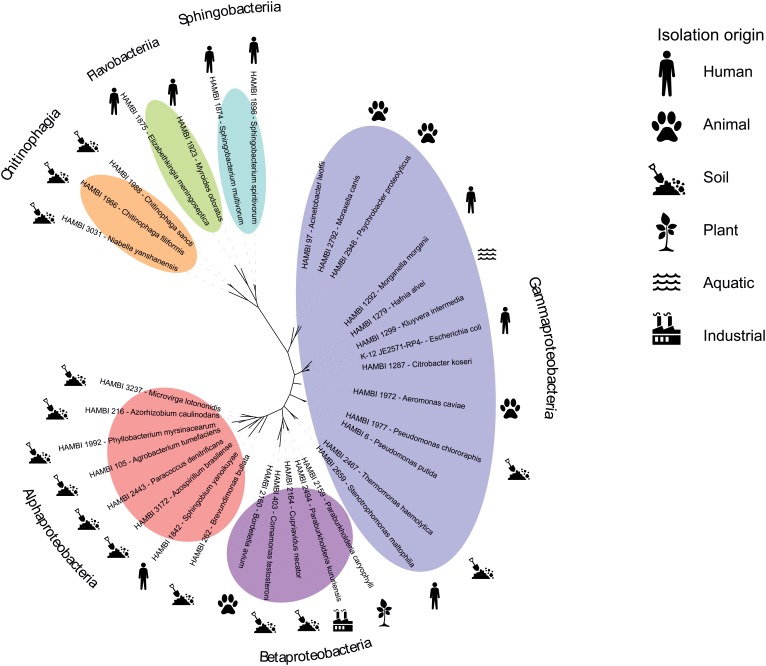
Phylogenetic relatedness and isolation origin of model community strains. Pictograms depict the isolation origin of the strains and the colored ovals their phylogenetic classes.

Previously, we performed a serial transfer experiment with an initial version of the model community consisting of 62 strains, including the *Escherichia coli* K-12 strain JE2571(RP4) harboring the multidrug resistance plasmid RP4 (Bradley, 1980; Cairns et al., 2018). The study demonstrates the ability of dozens of the strains to coexist in culture over 40 days, as well as a method for manipulating the level of spatial heterogeneity (biofilm mass) in community cultures. For tracking strain abundance over time in the study, we Sanger sequenced the near-full-length 16S rRNA gene sequences of the strains for use as a reference database for mapping high-throughput sequencing amplicons from experimental samples (Cairns et al., 2018). Based on these previous data, we refined the model community, removing strains with > 97% identity of 16S rRNA gene sequence with another strain in the community or uncertain strain identity. This resulted in a set of 33 gram-negative strains with confirmed culture collection identity that can be tracked at strain-level resolution through amplicon sequencing (**Supplementary Tables S1, S2**). The community was given the acronym HMC 33.1 (standing for HAMBI Mock Community containing 33 strains, version 1). The strains represent three classes in the phylum Proteobacteria (Alpha-, Beta- and Gammaproteobacteria) and three classes in the phylum Bacteroidetes (Chitinophagia, Flavobacteriia and Sphingobacteriia) (**Figure 1**). Each class contains a minimum of two strains, with the most representatives (13 strains) for the class Gammaproteobacteria. The strains have been isolated from diverse environments across the globe (**Supplementary Table S2**). Most of the strains originate from the soil, and the second highest group is human associated bacteria. Other sources include animals, aquatic environments, plants and the industry.

We further refined the co-culture medium to contain 1 g l^-1^ R2A solution and 0.5 g l^-1^ of cereal grass medium (Ward’s Science, St. Catharines, ON, Canada) in M9 salt solution. Cereal grass medium stock was prepared by autoclaving in dH_2_O and removing particulate matter by filtering through 5 μm.

To provide proof of concept for the coexistence of the strains in the medium, we performed a 48-day serial transfer experiment. The strains were grown separately for 4 days at 28°C with constant rotation at 50 rpm in the new medium and pooled together at equal volumes for the serial transfer experiment. The growth curves of strains when growth separately for 4 days are shown in **Supplementary Figure S1**. The stock community was stored in 30% glycerol at –80°C. The experiment was started by adding 10 μl of 100-fold diluted freeze-stored stock community to wells in a ABgene^TM^ 96 Well 2.2 ml Polypropylene Deepwell Storage Plate (Thermo Fisher Scientific, Waltham, MA, United States) containing 550 μl of medium. The community was grown at 28°C with constant rotation at 100 rpm. The experiment was maintained every 96 h by transferring 10% to fresh medium. To determine bacterial density, optical density values at 600 nm were obtained for undiluted grown cultures after each growth cycle (Tecan Infinite M200 well-plate reader), and samples were frozen in glycerol for further analysis.

### Phenotypic Analyses

Minimum inhibitory concentration (MIC) was determined for each strain for six antibiotics representing different antibiotic classes: ampicillin (class: penam), erythromycin (macrolide), kanamycin (aminoglycoside), nalidixic acid (fluoroquinolone), rifampicin (rifamycin), and streptomycin (aminoglycoside). For MIC determination, bacterial inoculum from an individual colony was suspended in M9 salt solution to 0.5 McFarland standard turbidity. Subsequently, 100 μl was spread-plated on 50% PPY agar medium containing a Liofilchem^®^ MIC test strip (Liofilchem, Italy) for a specific antibiotic. The MIC was interpreted according to manufacturer’s instructions after culturing at 28°C for 2 days, or for slow-growing strains, 6 days. Therefore, MICs were estimated in customized standard conditions rather than strain-specific optimal growth conditions or standard conditions recommended by the European Committee on Antimicrobial Susceptibility Testing (EUCAST) for clinical bacterial isolates (Kahlmeter et al., 2006), and the data might therefore not be comparable with clinical MIC data.

The ability of the strains to utilize 31 different carbon sources was assessed using the Biolog EcoPlate system (Biolog, Inc., Hayward, CA, United States), with the exception of HAMBI 2948 which grew poorly in experimental conditions. The experimental procedure was modified from MacLean et al. (2004). Strains were precultured in liquid PPY medium for 48 or 96 h, depending on strain growth ability, at 28°C with constant rotation. Subsequently, cells were spinned down, resuspended in M9 salt solution and nutrient-starved for 48–72 h. Starved cells were spinned down to remove any carryover nutrients in the supernatant, and resuspended in fresh M9 salt solution. Following 100-fold dilution into M9 salt solution, 150 μl of the culture was pipetted to wells in an EcoPlate (one EcoPlate per strain) containing three technical replicate wells for each carbon source and a negative control. The ability of a strain to utilize a particular carbon source was interpreted as a significantly higher optical density, based on a *t*-test, at 590 nm (measured with Tecan Infinite M200 well-plate reader) compared to the negative control after culturing for 7 days at 28°C.

### DNA Extraction and Sequencing for 16S rRNA Amplicon Analysis

DNA from three technical replicates from the original pooled bacterial community and three replicate communities from days 16, 32, and 48 in the serial transfer experiment was extracted using DNeasy 96 Blood & Tissue Kit (Qiagen, Hilden, Germany). DNA extraction was performed according to the manufacturer’s instructions using 400–600 μl of sample. DNA concentrations were measured using the Qubit^®^ 3.0 fluorometer (Thermo Fisher Scientific, Waltham, MA, United States).

Paired-end sequencing was performed using the Illumina MiSeq platform at the Institute for Molecular Medicine Finland (FIMM) amplifying the V3 and V4 regions of ribosomal RNA with Phusion High Fidelity PCR Master Mix (Thermo Fisher Scientific, Waltham, MA, United States). Reactions were done with a 2-step PCR method with primers carrying Illumina adapter tails (PCR1: forward 5′-ACACTCTTTCCCT ACACGACGCTCTTCCGATCTCTCCTACGGGAGGCAGCAG-3′, reverse 5-′AGACGTGTGCTCTTCCGATCTTCTRCGMATT YCACYKCTACAC-3′; PCR2: forward 5′-ACACTCTTTCCCT ACACGACGCTCTTCCGATCTGACTACHVGGGTATCTAATC C-3′, reverse 5′-AGACGTGTGCTCTTCCGATCTCCTACGGGN GGCWGCAG-3′). For PCR2, the primers are the same but the locus-specific part and adapter part have been paired vice versa to create more diversity in the final sequencing library. Products from PCR1 and PCR2 were pooled together and indexed with two Illumina P5/P7 index primers (every sample had their own unique combination).

PCR amplification was performed in a volume of 20 μl containing approx. 20 ng of sample DNA, 1 μl (5 μM) of each locus-specific primer (final concentration 0.25 μM), and 10 μl of 2 × Phusion High-Fidelity PCR Master Mix, and the reaction mix was brought to a final volume with laboratory grade water. The cycling conditions were as follows: 98°C for 30 s, 27 cycles of 98°C for 10 s, 62°C for 30 s, and 72°C for 15 s, with a final extension at 72°C for 10 min, followed by hold at 10°C.

Sample indexing was performed in a volume of 20 μl containing 1.5 μl (5 μM) of each index primer (final concentration 0.375 μM), 10 μl of 2 × Phusion High-Fidelity PCR Master Mix (Thermo Fisher Scientific, Waltham, MA, United States) and 1 μl of pooled PCR product. The reaction mix was brought to a final volume with laboratory grade water. The cycling conditions were as follows: 98°C for 30 s, 8 cycles of 98°C for 10 s, 65°C for 30 s, and 72°C for 20 s, with a final extension at 72°C for 5 min, followed by hold at 10°C.

After PCR, random samples were measured with LabChip GX Touch HT DNA High Sensitivity Reagent Kit (Perkin Elmer, Waltham, MA, United States) to check that the PCR was successful with the correct product size. The same run was repeated after indexing. Samples were pooled together in equal volumes and purified with Agencourt^®^ AMPure^®^ XP beads (Beckman Coulter, Brea, CA, United States) twice using 0.8 × volume of beads compared to the sample pool volume (40 μl). The ready amplicon library was diluted to 1:10 and 1:20 and quantified with the Agilent 2100 Bioanalyzer High Sensitivity DNA Analysis Kit (Agilent Genomics, Santa Clara, CA, United States). The 16S rRNA gene amplicon pool was sequenced with Illumina MiSeq System using the Illumina MiSeq Reagent Kit v3 600 cycles kit (Illumina, San Diego, CA, United States). The read length for the paired-end run was 2 × 300 bp.

### DNA Extraction and Sequencing for Whole-Genome Analysis

For this study, 16 of the 33 model community strains were whole-genome sequenced (**Table 1**). For the remaining 17 strains, 16 assembled genomes were obtained from the NCBI Reference Sequence Database (RefSeq) and 1 raw sequence dataset was obtained from the Joint Genome Institute Genomes OnLine Database (JGI GOLD; **Supplementary Table S1**). For the strains sequenced, DNA was extracted from 1 ml of liquid culture using the DNeasy 96 Blood & Tissue Kit (Qiagen, Hilden, Germany) according to manufacturer’s instructions. DNA concentrations were measured using the Qubit^®^ 3.0 fluorometer (Thermo Fisher Scientific, Waltham, MA, United States). High-throughput sequencing was performed by the Next Generation Sequencing Services, Institute for Molecular Medicine Finland (FIMM). For this, 2.5 ng of dsDNA was prepared according to the Nextera XT DNA Library Prep Kit Reference Guide (Illumina, San Diego, GA, United States) with the following modifications: All reactions were performed in half of the normal volume, and normalization was done according to the concentration measured on LabChip GX Touch HT (PerkinElmer, United States). 530–670 bp fragments were size selected from the pool using BluePippin (Sage Science, United States). Sequencing was performed with the Illumina HiSeq2500 system in the HiSeq high output mode using v4 kits (Illumina, San Diego, CA, United States). The read length for the paired-end run was 2 × 101 bp.

**Table 1:** Genome sequencing and assembly metrics.

Strain	Raw sequence yield (Gb)	Length (bp)	Coverage ±*SD*	% reads mapped	Largest contig (bp)	No. contigs	GC%	N50
HAMBI 6	0.76	6,425,774	88.4 ± 32.6	98.8	459,923	99	61.80	167,218
HAMBI 97	1.36	3,256,685	213 ± 63.1	97.4	201,770	162	41.41	32,660
HAMBI 105	0.79	5,387,408	110 ± 31.4	99.7	1,656,016	35	59.42	689,457
HAMBI 262	0.70	3,407,715	147 ± 49.7	98.6	203,726	88	67.02	103,424
HAMBI 1287	0.91	4,685,430	142 ± 40.2	99.7	540,344	31	53.81	453,729
HAMBI 1874	—^∗^	5,845,984	219 ± 36.4	94.9	337,658	94	39.90	117,696
HAMBI 1972	1.02	4,463,252	160 ± 54.3	99.2	404,185	61	61.77	179,321
HAMBI 1977	0.74	6,621,816	83.0 ± 31.5	99.4	675,539	57	63.08	355,467
HAMBI 1992	0.86	5,412,359	119 ± 35.6	99.6	544,523	57	59.33	262,180
HAMBI 2159	0.86	6,527,402	96.1 ± 30.2	99.0	590,902	59	64.73	222,449
HAMBI 2160	0.89	3,679,287	172 ± 49.3	99.2	544,789	48	61.65	278,194
HAMBI 2467	0.77	2,584,354	200 ± 48.3	98.5	519,133	29	70.03	174,888
HAMBI 2494	0.90	6,648,385	102 ± 32.1	98.9	781,864	68	65.45	259,448
HAMBI 2792	1.28	2,116,559	245 ± 22.5	95.7	118,108	103	45.03	51,649
HAMBI 2948	1.23	3,031,855	218 ± 55.3	100	1,099,313	22	42.82	657,030
HAMBI 3031	1.09	5,519,621	147 ± 55.8	99.9	1,089,344	28	42.70	633,609
HAMBI 3172	0.79	7,139,720	78.4 ± 29.8	97.7	230,491	186	68.98	71,229

*^∗^HAMBI 1874 was assembled but not sequenced in this study*.

### 16S rRNA Amplicon Analysis

Sequencing adapters were removed from unpaired sequence data using Cutadapt 1.12 (Martin, 2011), with the parameter –minimum-length 100. Sequence pairing was done with Pear 0.9.11 (Zhang et al., 2014). Quality was assessed before and after Cutadapt and Pear with FASTQC^1^, and further trimming was done with PRINSEQ (Schmieder and Edwards, 2011) using the parameters -trim_left 5 and -trim_right 40 to obtain better quality. Quality filtering was done with the USEARCH 10 (Edgar, 2013) command –fastq_filter with fastq_maxee setting 1.0 (to discard all reads with > 1.0 total expected errors). The command –usearch_global was used to align the sequences to the 16S rRNA fragment database of the strains with > 97% identity. The Shannon diversity index was computed using the USEARCH 10 command -alpha_div. Difference in community composition between days 16–48 (disregarding initial stock at time point 0 that might not represent viable cell counts for each strain) was assessed in R 3.4.0 (R Core Team, 2017) using permutational multivariate analysis of variance (PERMANOVA) as implemented in the adonis function in the vegan package (Anderson, 2001; Oksanen et al., 2016).

### Genome Assembly and Annotation

Prior to genome assembly, Cutadapt 1.12 (Martin, 2011) was used to remove sequencing adapters and quality trim sequence data, with the parameters -O 10 (minimum overlap for an adapter match) and -q 28 (quality cutoff for the 3′ end of each read). Sequence data quality before and after Cutadapt was assessed using FASTQC^1^ and multiQC (Ewels et al., 2016). Genome assembly was performed using SPAdes 3.11.1 (Bankevich et al., 2012) with default settings (i.e., no specified parameters since SPAdes performs estimation of e.g., k-mer sizes, coverage cutoff value and PHRED quality offset for input reads). Following assembly, contigs were filtered by minimum coverage 30 and minimum length 1000 bp. Genome assembly quality was assessed using QUAST 4.0 (Gurevich et al., 2013), and by mapping reads back to the assembly using Bowtie 2 with default settings (Langmead and Salzberg, 2012) combined with the Picard^2^ command CollectWgsMetrics performed for alignment files after sorting with SAMtools (Li et al., 2009) and marking duplicates with Picard (**Table 1**). Subsequently, all assemblies, including those obtained from databases, were annotated using Prokka 1.12 (Seemann, 2014), providing genus and species names, with the parameters –centre X, –compliant, and –usegenus.

### Phylogenetic Analysis

We identified and aligned genes conserved among all community members using Roary 3.8.0-0 (Page et al., 2015) with minimum BLASTp identity set to 65% and protein group limit set to 200,000 to account for high diversity in the community. The core gene alignment, consisting of four ribosomal proteins (*rplN*, *rpmA*, *rpsL*, and *rpsS*) and the ATP synthase subunit beta (*atpD*), was used to create a phylogenetic tree with PhyML (Guindon et al., 2010), visualized in iTol (Letunic and Bork, 2016).

### Characterization of Genomic Features

To generate comprehensive genomic metadata for model community strains, the genome assemblies were scanned for genomic elements using several tools, with default parameters unless otherwise specified. The Metabolic And Physiological potentiaL Evaluator (MAPLE) 2.3.0 (Takami et al., 2016) was used to map genes in assemblies to functional modules as defined by the Kyoto Encyclopedia of Genes and Genomes (KEGG) and for calculating module completion ratios (MCR). Genomic islands, i.e., large genomic regions assumed to have horizontal origins, were predicted using IslandViewer 4 (Bertelli et al., 2017). For strains other than *E*. *coli* K-12, HAMBI 216, HAMBI 1923 and HAMBI 2659 which have closed genomes, genomic island predictions were obtained for incomplete genomes with contigs ordered against closely related reference genomes (**Supplementary Table S3**) using the Mauve contig orderer (Rissman et al., 2009; Bertelli et al., 2017).

Plasmid-derived sequences were predicted using cBar 1.2 (Zhou and Xu, 2010) and PlasmidFinder 1.3 (Carattoli et al., 2014), which was implemented through ABRicate^3^ using databases dated 2018-01-02. In addition, for genomes assembled for this study, plasmidSPAdes (Antipov et al., 2016) implemented in SPAdes 3.11.1 (Bankevich et al., 2012) was used, with assignment of plasmid annotation to > 1000 bp regions in genome assembly contigs displaying 100% BLASTn (Altschul et al., 1990) identity with plasmid assemblies generated by plasmidSPAdes.

Antibiotic resistance and virulence genes were predicted with ABRicate (https://github.com/tseemann/abricate, databases dated 2018-01-02) using ResFinder 3.0 (Zankari et al., 2012), the comprehensive antibiotic resistance database (CARD) (Jia et al., 2017), and the virulence factors database (VFDB) (Chen et al., 2016), discarding hits with proportion of gene covered < 50%. Prophages, integrons, insertion sequences, and clustered regularly interspaced short palindromic repeats (CRISPR) were predicted using the PHAge Search Tool – Enhanced Release (PHASTER; accessed 2018-01-08) (Arndt et al., 2016), Integron Finder 1.5.1 (Cury et al., 2016), ISEScan 1.5.4.3 (Xie and Tang, 2017) and CRISPRFinder (accessed 2018-02-01) (Grissa et al., 2007), respectively. Notably, in the absence of further validation steps, plasmid predictions obtained with cBar or plasmidSPAdes have low accuracy (Arredondo-Alonso et al., 2017), and genomic island predictions for draft level genomes may contain errors (Bertelli et al., 2017), and the predicted loci must therefore be considered as regions of interest rather than high-confidence predictions.

## Results

### Genomic Composition

**FIGURE 2:**
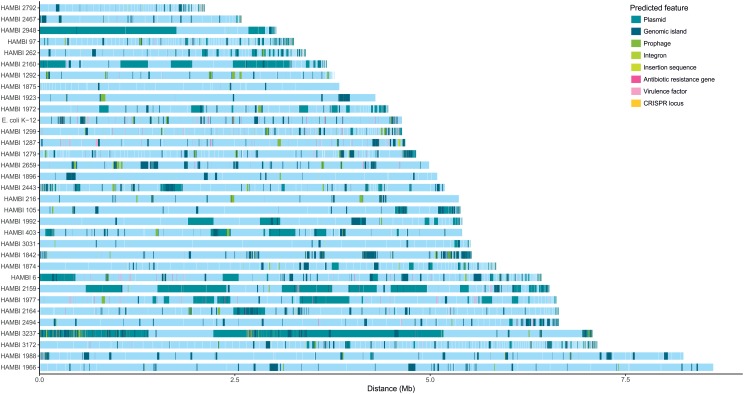
Genomic features of model community strains. *E*. *coli* K-12, HAMBI 216, HAMBI 1923 and HAMBI 2659 are closed genomes composed of a single chromosome. Other genomes are composed of blocks of adjacent contigs whose order may not reflect genomic position. Contigs are ordered by size for genomes assembled in this study and by order of appearance in assembly file for genomes obtained from databases. Predicted features are depicted by colored blocks along the genome. These include plasmid-derived sequences, genomic islands, prophages, integrons, insertion sequences, antibiotic resistance genes, virulence factors, and CRISPR loci.

The strains display high variability in genome size and genomic content, including the resistome, mobilome and functionome (**Figure 2**). Antibiotic resistance and virulence genes are particularly prevalent among strains in the Gammaproteobacteria class (**Figures 1**, **2**). Genomic elements associated with horizontal gene transfer, including plasmids, genomic islands, prophages, integrons, and insertion sequences, occur frequently among the strains, as do CRISPR arrays that might be associated with CRISPR/Cas systems conferring adaptive immunity against mobile genetic elements.

### Metabolic and Physiological Characteristics

**FIGURE 3:**
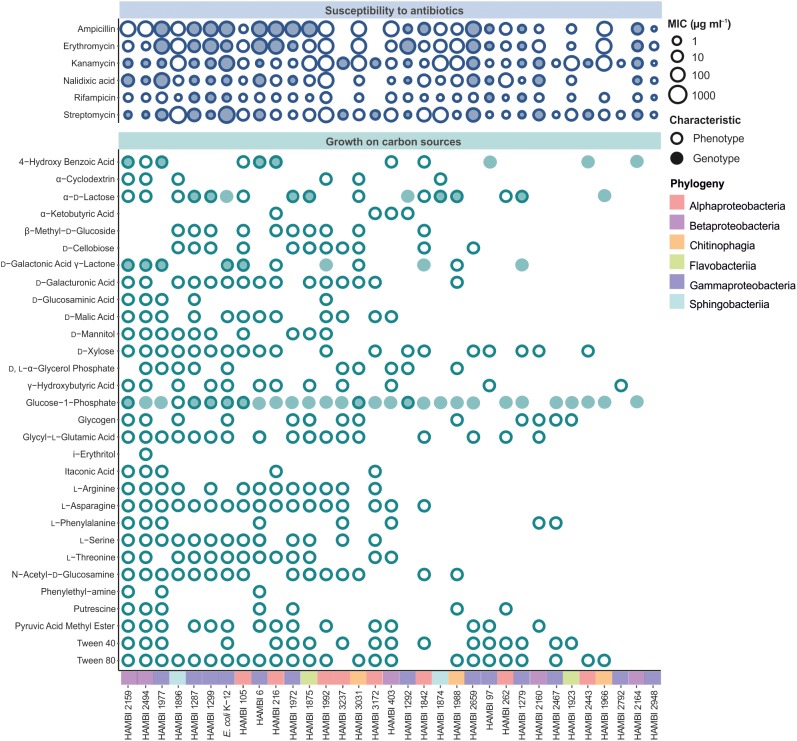
Functional characteristics of model community strains. Both phenotypic (empty circle) and bioinformatically predicted (filled circle) susceptibility to antibiotics (upper panel) and ability to grow on a carbon source (lower panel) are shown. Matching between carbon source utilization phenotypes and functions predicted from genome sequences was only performed for 4-hydroxy benzoic acid, glucose-1-phosphate, D-galactonic acid γ-lactone, D-galacturonic acid and α-D-lactose where filled circles represent the presence of genomic modules for benzoic acid, glucose, galactonic acid, galacturonic acid and lactose metabolism, respectively. The strains are depicted in descending order of total number of carbon sources utilized. MIC, minimum inhibitory concentration.

The strains display diverse antibiotic susceptibility and carbon source utilization phenotypes (**Figure 3**). Antibiotic resistance phenotypes are variably associated with the presence of bioinformatically predicted resistance factors to the antibiotic class. The number of carbon sources on which the strains can grow displays high variability, with a maximum of 25 out of 31 carbon sources (HAMBI 2159 and 2494, which both belong to the genus *Paraburkholderia*), indicating large differences between strains in metabolic capacity and fastidiousness. Furthermore, the presence of genomic functional modules for benzoic acid, galactonic acid, galacturonic acid or lactose metabolism is frequently associated with the ability of a strain to grow with a corresponding compound as the sole carbon source (**Figure 3**).

**FIGURE 4:**
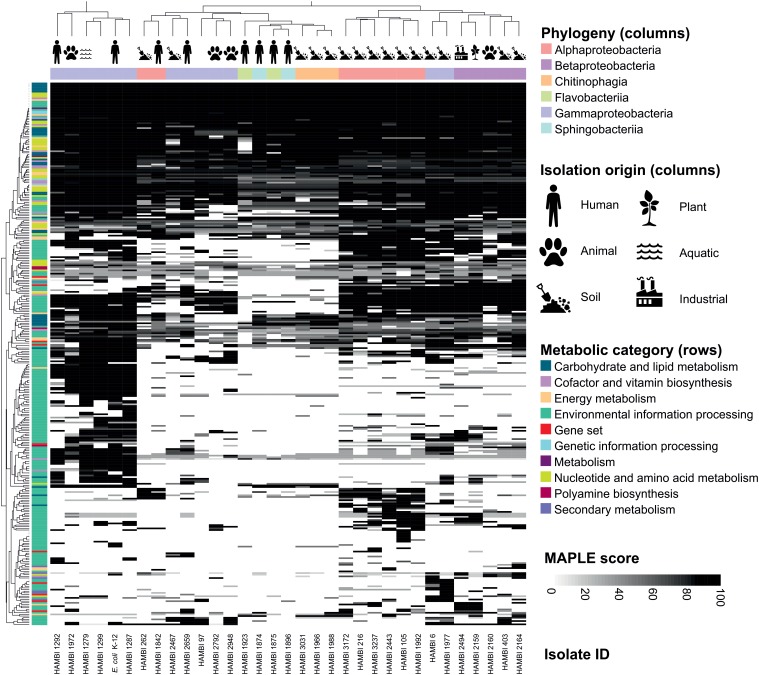
Metabolic pathways of model community strains. The pathways were predicted from genome sequences using MAPLE. Pathway completeness (MAPLE score) is depicted as shades of gray in a heat map organized by the phylogenetic class and isolation origin of model community strains (columns) and by the presence of metabolic pathways predicted by MAPLE (rows). The metabolic characteristics display hierarchical clustering by the phylogenetic class and isolation origin of the isolates.

The metabolic characteristics predicted from the genome sequences by MAPLE display hierarchical clustering by phylogenetic classes of the isolates as well as by their isolation origin (**Figure 4**). Soil isolates typically cluster separately from isolates from humans and animals. Most of this clustering is driven by metabolic functions related to environmental information processing. Core metabolic pathways such as the tricarboxylic acid cycle are present across all isolates while specialized metabolic features, such as utilization of less common carbohydrates, exhibit differences in their distribution across the isolates.

### Coexistence of Strains in Co-culture

**FIGURE 5:**
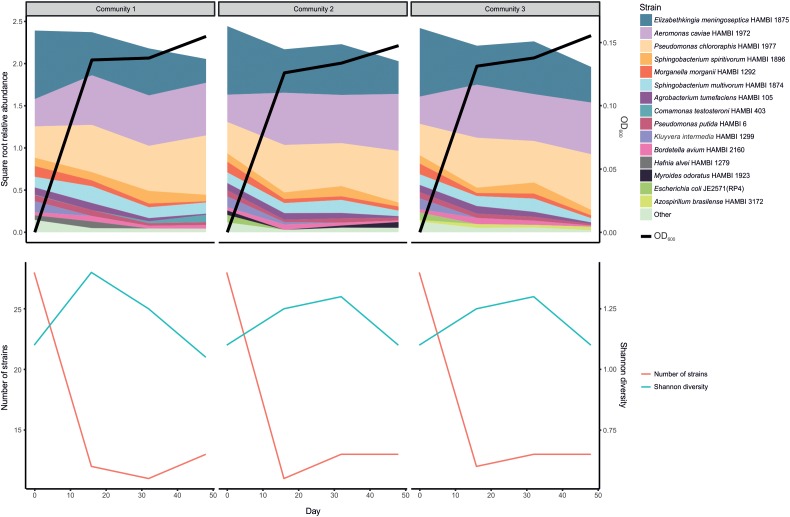
Persistence of strains in model community when serially transferred for 48 days (three replicate communities). The upper panel shows the relative abundance of the 15 most abundant taxa and a proxy for total cell density (optical density, OD, at 600 nm). The lower panel shows two diversity indices, richness (strain count) and the Shannon index.

Serially transferring the 33 strain community for 48 days seemingly led to a loss of over half of the strains within the first 16 days, although the initial number of viable strains is uncertain owing to different growth dynamics of individual strains prior to initiating the experiment (**Supplementary Figure S1** and **Figure 5**). After these transient dynamics, there was only a marginally significant change in community composition over time between days 16 and 48 (PERMANOVA: *F*_1,7_ = 3.018, *r^2^* = 0.301, *p* = 0.074). Furthermore, the apparent initial loss in richness (strain count) was accompanied by an increase in evenness, seen as a relatively small temporal change in Shannon diversity, which accounts for both factors. There were three dominant strains: *Elizabethkingia meningoseptica* HAMBI 1875, *Aeromonas caviae* HAMBI 1972 and *Pseudomonas chlororaphis* HAMBI 1977. However, the degree to which the dominance of these strains is caused by their relative abundance or viability in the input community (strain mix) or competitive ability during co-culture is uncertain.

## Discussion and Conclusion

We developed a multispecies synthetic bacterial community, which was characterized at the genomic and phenotypic levels, revealing high diversity in the resistome, mobilome and functionome of the community. Furthermore, we demonstrated the utility of the community by showing coexistence of a large proportion (14/33) of the strains in co-culture over serial transfer for 48 days. These observations indicate that the community is suitable for use as a cultivated model community to ask a wide range of biological questions.

Along with characterizing the community, we produced comprehensive genomic and phenotypic metadata for the community members. This facilitates future mechanistic work with the community. For instance, our pipeline allows the use of amplicon sequencing to track community composition over time at strain-level resolution. Moreover, the genome assemblies provide a reference database for (meta)genomic and (meta)transcriptomic studies. Isolation of individual colonies combined with colony PCR, in turn, allows the rapid identification of substrains possessing mutations or horizontal gene transfer events of interest.

Our model community is composed of diverse bacterial strains isolated from different environments and hence does not mimic any specific natural community. Therefore, the community is more suited to study general questions about ecology and evolution such as community assembly and response to environmental perturbations rather than to explain patterns in any particular natural microbial community (Wolfe, 2018). The ability of the community to answer general questions may be limited by the potential absence of focal taxonomic groups or high-order interactions that occur in more complex communities. The community is composed of bacteria alone, while interactions between bacteria and fungi, protozoa or bacteriophages often play a key role in natural microbial communities. In a separate study, however, we have introduced a method for incorporating protozoa in the community (Cairns et al., 2018).

The utility of the 33-strain model community and its predecessor is demonstrated in the current and previous work by us (Cairns et al., 2018). Here we present the community and a collection of methods and metadata to the scientific community. A previous version of the community has already been successfully used for tracking the mobility of antibiotic resistance genes (Cairns et al., 2018), and as prominent cases of future use we envision, for instance, replicated ecosystem microcosms to explore the trajectories of ecosystem composition and genetic structure in response to various environmental perturbations. We also envision the community as an efficient spike-in control to increase the statistical rigor of high-throughput microbial single cell assays such as epicPCR (Spencer et al., 2016) or as a test system for validating metagenome assemblers.

## Data Availability

Raw fastq files and genome assemblies for the genomes sequenced or assembled in this study (*N* = 17) have been deposited in the National Center for Biotechnology Information (NCBI) Sequence Read Archive (SRA) and GenBank, respectively, under the BioProject Accession Number PRJNA476209. Accession numbers for raw fastq files or genome assemblies obtained for this study from databases are listed in **Supplementary Table S1**. Previously Sanger sequenced near-full-length 16S rRNA gene sequences for all strains are available in the European Nucleotide Archive (ENA) under the Accession Number PRJEB21728 (https://www.ebi.ac.uk/ena/data/view/PRJEB21728). The complete sequence for the plasmid RP4 harbored by *E*. *coli* K-12 JE2571(RP4) is available from GenBank under the accession BN000925 (Pansegrau et al., 1994). Genome annotations, including those obtained in this study for previously sequenced or assembled genomes, are available upon request.

The following community metadata is available via Dryad (doi: 10.5061/dryad.53b6n5f): Genomic predictions for functional modules obtained using MAPLE 2.3.0 (Takami et al., 2016); genomic feature data collated into a single data frame, using custom R scripts (R Core Team, 2017), displaying strain, contig, locus, and genomic feature information; antibiotic MICs; and carbon source utilization phenotypes. Raw output files for each strain from all genomic feature prediction tools are available upon request.

The model community strains, except for *E. coli* K-12 strain JE2571(RP4), are available individually from the HAMBI culture collection at the University of Helsinki, Finland (http://www.helsinki.fi/hambi/). For further requests concerning the use of the model community, please contact the corresponding author.

## Author Contributions

JC and TH designed the experiments. RJ contributed to phenotypic measurements, proof of concept experiments, and performed sequence analyses for 16S rRNA amplicon data. TH, JH, and MV provided sequencing resources. JC and JH designed the sequence analyses. JC performed sequence analyses for whole-genome data and drafted the manuscript. JC, MT, and RJ contributed to data visualization. RJ, JH, MT, MV, and TH edited and commented on the manuscript. All authors gave final approval for publication and accept accountability for the content and work performed.

## Conflict of Interest Statement

The authors declare that the research was conducted in the absence of any commercial or financial relationships that could be construed as a potential conflict of interest.

## Footnotes

1 http://www.bioinformatics.babraham.ac.uk/projects/fastqc

2 http://broadinstitute.github.io/picard

3 https://github.com/tseemann/abricate
