# Supplementary material for: Construction and Characterization of Synthetic Bacterial Community for Experimental Ecology and Evolution
Source: Front Genet. 2018 Aug 14;9:312. doi: 10.3389/fgene.2018.00312 (PMC6102323; doi:10.3389/fgene.2018.00312)
Supplement: Supplementary file 1 [file Data_Sheet_1.pdf]

Supplementary Material for

Construction and Characterization of Synthetic Bacterial Community for Experimental Ecology and Evolution

Johannes Cairns, Roosa Jokela, Jenni Hultman, Manu Tamminen, Marko Virta and Teppo Hiltunen

Correspondence to: teppo.hiltunen@helsinki.fi

This PDF file includes:

Figure S1

Tables S1 to S3

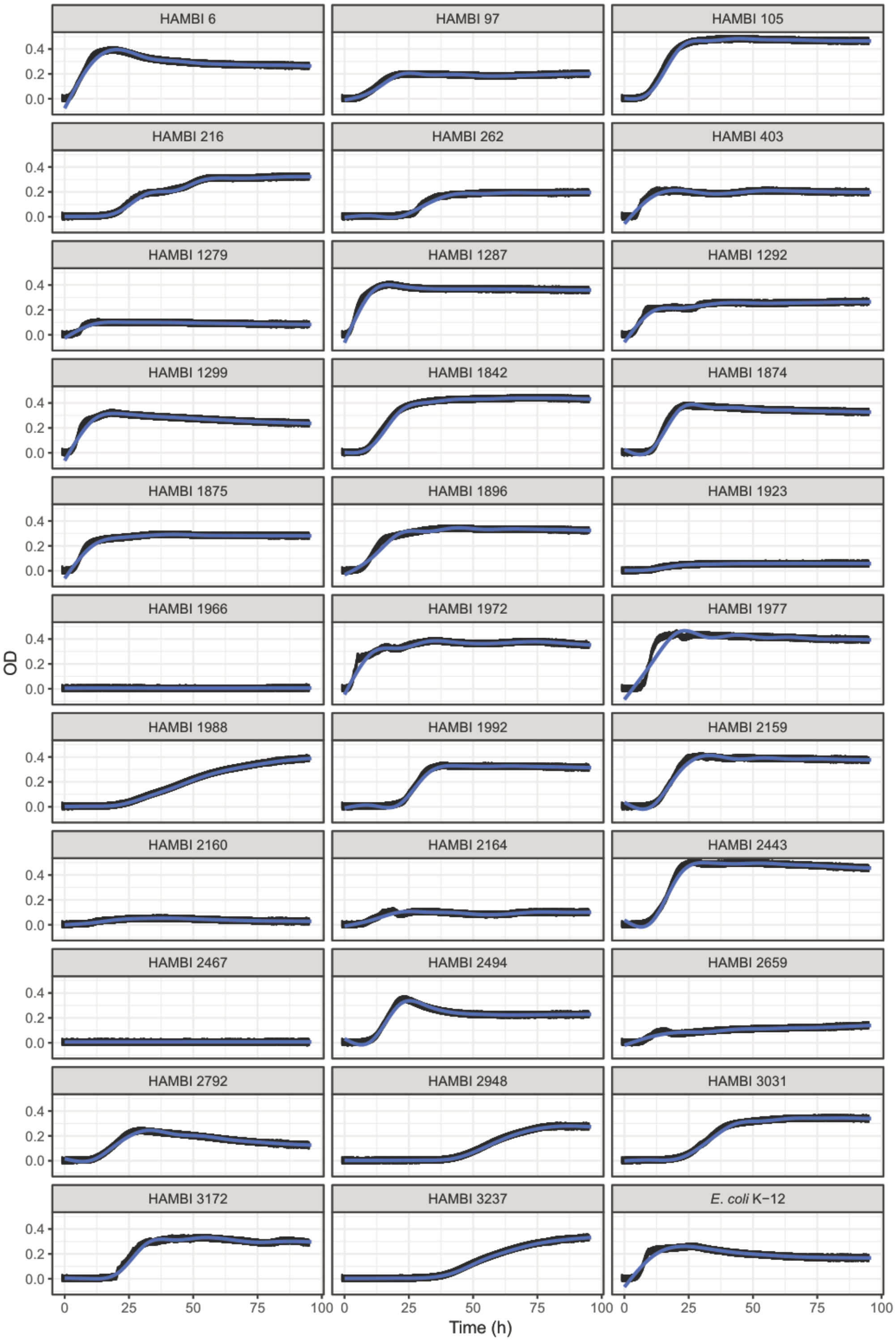

Figure S1. Growth curves of HMC 33.1 strains cultured separately for 96 h in experimental medium. Each curve (black line) is based on four technical replicates, and a smoother (blue line) modeling the shape of the curve using a general additive model is also indicated. Measurements were performed with the Bioscreen C well-plate reader (Labsystems Oy, Helsinki, Finland) on clonal cultures diluted to low optical density (OD), using a wideband filter to measure $OD_{420–580\ nm}$ at 5 min intervals.

Table S1. Composition of HAMBI Mock Community (HMC) 33.1 constructed for this study.

Strain	Other ID*	Genome accession	Database**
Acinetobacter johnsonii strain HAMBI 97	ATCC 9036	—	—
Aeromonas caviae strain HAMBI 1972	NCIMB 13016	—	—
Agrobacterium tumefaciens strain HAMBI 105	ATCC 4718	—	—
Azorhizobium caulinodans strain HAMBI 216	ORS 571 T	GCF 000010525	RefSeq
Azospirillum brasilense strain HAMBI 3172	LMG 4376	—	—
Bordetella avium strain HAMBI 2160	ATCC 35086 T	—	—
Brevundimonas bullata strain HAMBI 262	ATCC 4278 T	—	—
Chitinophaga filiformis strain HAMBI 1966	DSM 527 T	—	—
Chitinophaga sancti strain HAMBI 1988	DSM 784 T	GCF 900119105	RefSeq
Citrobacter koseri strain HAMBI 1287	CUETM 77.4	—	—
Comamonas testosteroni strain HAMBI 403	ATCC 11996 T	GCF 000241525	RefSeq
Cupriavidus necator strain HAMBI 2164	DSM 1105	GCF 001592245	RefSeq
Elizabethkingia meningoseptica strain HAMBI 1875	NCTC 10016	GCF 000367325	RefSeq
Escherichia coli K-12 strain JE2571(RP4)	—	GCF 000005845	RefSeq
Hafnia alvei strain HAMBI 1279	ATCC 13337 T	GCF 000735375	RefSeq
Kluyvera intermedia strain HAMBI 1299	ATCC 33110 T	GCF 001598315	RefSeq
Microvirga lotononidis strain HAMBI 3237	WSM 3557	GCF 000262405	RefSeq
Moraxella canis strain HAMBI 2792	—	—	—
Morganella morganii strain HAMBI 1292	ATCC 25830	GCF 001598895	RefSeq
Myroides odoratus strain HAMBI 1923	NCTC 11036	GCF 000243275	RefSeq
Niabella yanshanensis strain HAMBI 3031	CCBAU 05354	—	—
Paraburkholderia caryophylli strain HAMBI 2159	DSM 50341 T	—	—
Paraburkholderia kururiensis strain HAMBI 2494	—	—	—
Paracoccus denitrificans strain HAMBI 2443	NCTC 11036	GCF 900100045	RefSeq
Phyllobacterium myrsinacearum strain HAMBI 1992	LMG 2(t2) T	—	—
Pseudomonas chlororaphis strain HAMBI 1977	ATCC 17415	—	—
Pseudomonas putida strain HAMBI 6	ATCC 11172	—	—
Psychrobacter proteolyticus strain HAMBI 2948	DSM 13887 T	—	—
Sphingobacterium multivorum strain HAMBI 1874	NCTC 11343	Gp0013291	JGI GOLD
Sphingobacterium spiritivorum strain HAMBI 1896	NCTC 11386	GCF 000143765	RefSeq
Sphingobium yanoikuyae strain HAMBI 1842	DSM 7462 T	GCF 000315525	RefSeq
Stenotrophomonas maltophilia strain HAMBI 2659	ATCC 13637	GCF 000742995	RefSeq
Thermomonas haemolytica strain HAMBI 2467	DSM 13605 T	—	—

*T = type strain.
**RefSeq accessions are for genome assemblies, and the JGI GOLD accession is for raw sequence data (fastq) files.
Culture collection acronyms: ATCC = American Type Culture Collection; CCBAU = Culture Collection of Beijiing Agricultural University, People's Republic of China; CUETM = Collection de l'Unité d'Écotoxicologie Microbienne, Institut National de la Recherche Agronomique (INRA), France; DSM = DSMZ-Deutsche Sammlung von Mikroorganismen und Zellkulturen GmbH, Germany; HAMBI = University of Helsinki Culture Collection (UHCC); LMG = Collection of the Laboratorium voor Microbiologie en Microbiele Genetica, Rijksuniversiteit, Belgium; NCIMB = National Collection of Industrial and Marine Bacteria, National Collections of Industrial, Food and Marine Bacteria, UK; NCTC = National Collection of Type Cultures, Central Public Health Laboratory, UK.

Table S2. Source of isolation of HMC 33.1 strains.

Strain	Source of isolation
Acinetobacter johnsonii strain HAMBI 97	NA
Aeromonas caviae strain HAMBI 1972	Epizootic of young guinea pigs, KY, USA
Agrobacterium tumefaciens strain HAMBI 105	Soil, USA
Azorhizobium caulinodans strain HAMBI 216	*Sesbania rostrata* stem nodules, North Senegal
Azospirillum brasilense strain HAMBI 3172	*Oryza sativa* root, Bogor, Indonesia
Bordetella avium strain HAMBI 2160	Respiratory tract of turkey poults, West Germany
Brevundimonas bullata strain HAMBI 262	Soil
Chitinophaga filiformis strain HAMBI 1966	Soil, Samoa
Chitinophaga sancti strain HAMBI 1988	Soil
Citrobacter koseri strain HAMBI 1287	NA
Comamonas testosteroni strain HAMBI 403	Soil, Berkeley
Cupriavidus necator strain HAMBI 2164	Garden soil
Elizabethkingia meningoseptica strain HAMBI 1875	Spinal fluid from premature infant, MA, USA
Escherichia coli K-12 strain JE2571(RP4)	Human stool, Stanford, USA
Hafnia alvei strain HAMBI 1279	NA
Kluyvera intermedia strain HAMBI 1299	Surface water, France
Microvirga lotononidis strain HAMBI 3237	Nitrogen-fixing nodule of *Lotononsis angolensis*, Chibala, Zambia
Moraxella canis strain HAMBI 2792	Dog's beard, Finland
Morganella morganii strain HAMBI 1292	Human with summer diarrhea, London, UK
Myroides odoratus strain HAMBI 1923	Feces of patient with intestinal infection, Europe
Niabella yanshanensis strain HAMBI 3031	Soybean rhizosphere, Hebei, China
Paraburkholderia caryophylli strain HAMBI 2159	Carnation, USA
Paraburkholderia kururiensis strain HAMBI 2494	Printing paper machine, Finland
Paracoccus denitrificans strain HAMBI 2443	Garden soil, Delft, Netherlands
Phyllobacterium myrsinacearum strain HAMBI 1992	Leaf nodule of *Ardisia crispa* in tropical greenhouse, Germany
Pseudomonas chlororaphis strain HAMBI 1977	Farm soil, Peoria, IL, USA
Pseudomonas putida strain HAMBI 6	NA
Psychrobacter proteolyticus strain HAMBI 2948	Stomach specimen of Antarctic krill *Euphausia superba*
Sphingobacterium multivorum strain HAMBI 1874	Human clinical specimen, spleen, WA, USA
Sphingobacterium spiritivorum strain HAMBI 1896	Human, intra-uterine, KS, USA
Sphingobium yanoikuyae strain HAMBI 1842	Clinical specimen
Stenotrophomonas maltophilia strain HAMBI 2659	Oropharyngeal region of patient with mouth cancer
Thermomonas haemolytica strain HAMBI 2467	Kaolin slurry, Finland

Table S3. Reference genomes used for genomic island prediction with incomplete genomes.

Strain	Reference genome in IslandViewer 4 database
Acinetobacter johnsonii strain HAMBI 97	*Acinetobacter johnsonii* strain XBB1
Aeromonas caviae strain HAMBI 1972	*Aeromonas* sp. strain CU5
Agrobacterium tumefaciens strain HAMBI 105	*Agrobacterium tumefaciens* strain Ach5
Azorhizobium caulinodans strain HAMBI 216	NA (complete genome)
Azospirillum brasilense strain HAMBI 3172	*Azospirillum brasilense* strain Az39
Bordetella avium strain HAMBI 2160	*Bordetella avium* strain 197N
Brevundimonas bullata strain HAMBI 262	*Brevundimonas* sp. strain DS20
Chitinophaga filiformis strain HAMBI 1966	*Chitinophaga pinensis* strain DSM 2588
Chitinophaga sancti strain HAMBI 1988	*Chitinophaga pinensis* strain DSM 2588
Citrobacter koseri strain HAMBI 1287	*Citrobacter koseri* strain ATCC BAA-895
Comamonas testosteroni strain HAMBI 403	*Comamonas testosteroni* strain CNB-2
Cupriavidus necator strain HAMBI 2164	*Cupriavidus necator* strain N-1
Elizabethkingia meningoseptica strain HAMBI 1875	*Elizabethkingia meningoseptica* strain G4076
Escherichia coli K-12 strain JE2571(RP4)	NA (complete genome)
Hafnia alvei strain HAMBI 1279	*Hafnia alvei* strain FB1
Kluyvera intermedia strain HAMBI 1299	*Enterobacter* sp. strain 638
Microvirga lotononidis strain HAMBI 3237	*Microvirga ossetica* strain V5/3M
Moraxella canis strain HAMBI 2792	*Moraxella ovis* strain 199/55
Morganella morganii strain HAMBI 1292	*Morganella morganii* subsp. Morganii strain KT
Myroides odoratus strain HAMBI 1923	NA (complete genome)
Niabella yanshanensis strain HAMBI 3031	*Niabella soli* strain DSM 19437
Paraburkholderia caryophylli strain HAMBI 2159	*Paraburkholderia* sp. strain SOS3
Paraburkholderia kururiensis strain HAMBI 2494	*Paraburkholderia* sp. strain SOS3
Paracoccus denitrificans strain HAMBI 2443	*Paracoccus denitrificans* strain PD1222
Phyllobacterium myrsinacearum strain HAMBI 1992	*Phyllobacterium* sp. strain Tri-48
Pseudomonas chlororaphis strain HAMBI 1977	*Pseudomonas chlororaphis* strain PA23
Pseudomonas putida strain HAMBI 6	*Pseudomonas putida* strain KT2440
Psychrobacter proteolyticus strain HAMBI 2948	*Psychrobacter* sp. strain G
Sphingobacterium multivorum strain HAMBI 1874	*Sphingobacterium* sp. strain 21
Sphingobacterium spiritivorum strain HAMBI 1896	*Sphingobacterium* sp. strain 21
Sphingobium yanoikuyae strain HAMBI 1842	*Sphingomonas* sp. strain JJ-A5
Stenotrophomonas maltophilia strain HAMBI 2659	NA (complete genome)
Thermomonas haemolytica strain HAMBI 2467	NA (closely related reference not found)
